# Occupational Allergic Contact Dermatitis to Ethylhexylglycerin in an Alcohol‐Based Hand Disinfectant

**DOI:** 10.1111/cod.14802

**Published:** 2025-04-17

**Authors:** Richard Brans, Christoph Skudlik

**Affiliations:** ^1^ Institute for Interdisciplinary Dermatologic Prevention and Rehabilitation (iDerm) at the Osnabrück University Osnabrück Germany; ^2^ Department of Dermatology, Environmental Medicine and Health Theory Osnabrück University Osnabrück Germany

**Keywords:** 3‐(2‐ethylhexyloxy)propane‐1,2‐diol, allergic contact dermatitis, CAS no. 70445–33‐9, case report, disinfectant, ethylhexylglycerin, occupational, octoxyglycerin

Ethylhexylglycerin rarely causes allergic contact dermatitis (ACD) which mainly is related to cosmetics [1, 2, 3, 4]. Here, we present a case of occupational ACD caused by ethylhexylglycerin in an alcohol‐based hand disinfectant.

## 1 Case Report

A 30‐year‐old woman without a history of atopy had worked as a surgical assistant for 5 years before developing severe dermatitis on her lower arms, which evolved within a couple of days. As usual, she had performed surgical disinfections of her hands and lower arms several times a day using one of the following alcohol‐based hand disinfectants provided at work: Desmanol pure, Desmanol care, or Desderman (all from Schülke & Mayr, Norderstedt, Germany). Allocation of the skin lesions to one of them was not possible, as she had alternated their use. Afterwards, she disinfected her lower arms with different alcohol‐based disinfectants but had the impression that she would not tolerate any of them. Therefore, she was exempted from surgical assistance and thus from performing surgical disinfections of her lower arms.

**FIGURE 1:**
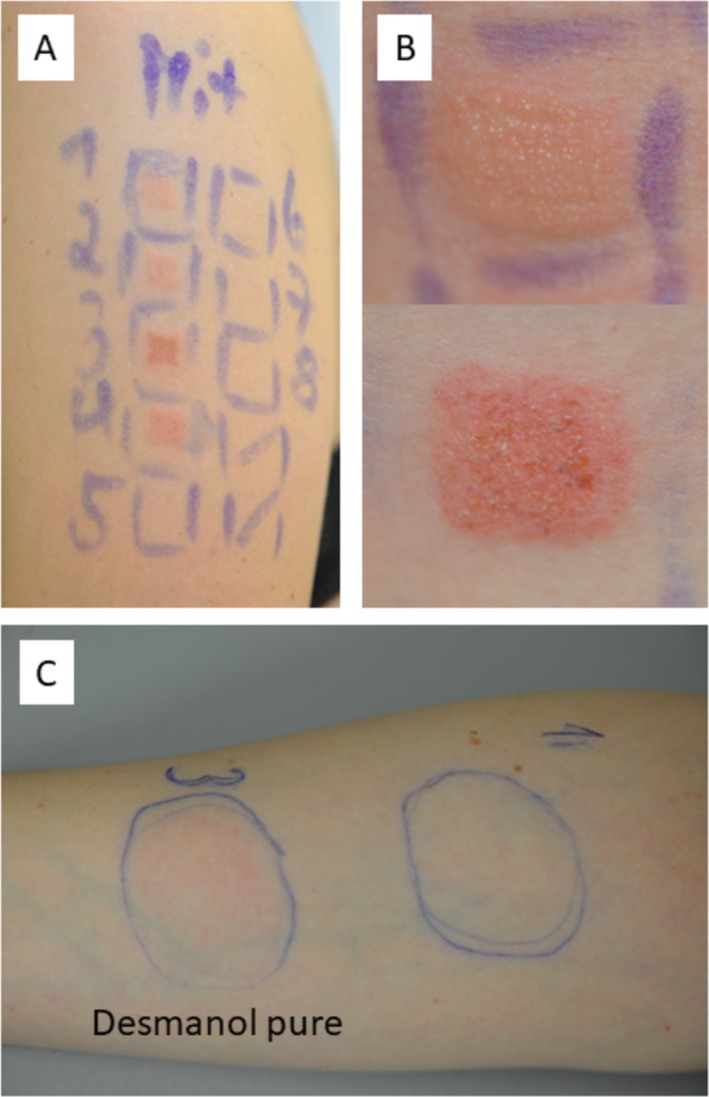
(A) Patch test reading on day (D)3 for the four alcohol‐based hand disinfectants with positive reactions to Desderman ‘as is’ (1), Desderman 50% aqu. (2), Desmanol Pure ‘as is’ (3), and Desmanol Pure 50% aqu. (4); (B) positive patch test to ethylhexylglycerin 5% pet. on D2 (top) and D3 (bottom); (C) positive Repeated Open Application Test (ROAT) to Desmanol pure ‘as is’ on D3.

A patch test was performed and read according to the guidelines of the German Contact Dermatitis Research Group (DKG) [2] using the DKG baseline series and the DKG series for ‘ingredients of topical preparations’, ‘preservatives’, ‘rubber’, ‘disinfectants’, the patient's own three hand disinfectants, and another product (Descoderm, Dr. Schumacher, Malsfeld, Germany) which according to the patient had also caused skin lesions on her lower arms. The commercial patch test preparations were purchased from SmartPractice Europe (Greven, Germany). The hand disinfectants were patch tested ‘as is’ and in a 50% aqueous dilution with an occlusion time of one day (D). The shorter occlusion time was chosen as the patient had reported severe skin reactions after open application of the disinfectans during regular use. All other patches were removed on D2 and readings were done on (D1), D2, D3, and D4. Strong positive patch tests reactions to ethylhexylglyerin 5% pet. (++) were observed on D2, D3 and D4. No positive reaction to any of the other commercial patch test preparations, including povidone iodine 10% aq., occurred, but the patient developed positive reactions to both tested concentrations of Desmanol pure (D2 +, D3/D4: ++) and Desderman (D2‐D4: +) (Figure 1). During patch testing, a Repeated Open Application Test (ROAT) with all four hand disinfectants ‘as is’ was performed on the inside of her lower arms. Starting from D2, she developed a positive reaction to Desmanol pure. No reactions to the other three hand disinfectants occurred until D4. The patient was advised to continue the ROAT with Descoderm at home for up to ten days and it remained negative. Upon request, the manufacturer confirmed that Desmanol pure contains ethylhexylgycerin. No complete list of its constituents was publicly available or provided by the manufacturer stating that Desmanol pure is registered as biocidal product for which it is not mandatory to reveal all constituents. The safety data sheet only indicated the presence of 2‐propanol and tetradecanol. Interestingly, Desderman from the same manufacturer is registered as a medicinal product and a list of all its constituents is publicly available. Apart from ethanol and 2‐propanol, the product contains butanone, isopropylmyristate, (hexadecyl/octadecyl) (2‐ethylhexanoate), polyvinylpyrrolidon K 30, sorbitol and purified water. The manufacturer denied provision of these ingredients for supplemental patch testing. Sensitization to one of the ingredients is possible, but without any break‐down testing we cannot exclude an irritant patch test reaction to Desderman as the ROAT until D4 remained negative, being aware that a longer duration of the ROAT would have been desirable to further exclude or confirm an allergic reaction [5]. As Descoderm does not contain ethylhexylglycerin or glycerin/glycerol and was well tolerated both in the patch test and in the ROAT, the patient was advised to use this product for skin disinfections in the future.

## 2 Discussion

Ethylhexylglycerin (syn. Octoxyglycerin, 3‐(2‐ethylhexyloxy)propane‐1,2‐diol, CAS no. 70445–33‐9) is an alkyl glyceryl ether formed from 2‐ethylhexanol and glycerin [1] Since its introduction under the trade name Sensiva SC 50 (Schülke & Mayr, Norderstedt, Germany) in 1992, it has been used as a surfactant, emollient and because of its deodorising and antimicrobial properties in various cosmetics [3] It was shown to enhance the efficacy of preservatives and other antimicrobial agents, such as chlorhexidine gluconate and benzalkonium chloride in alcohol‐based hand disinfectants [6, 7]. The prevalence of contact allergy to ethylhexylglycerin in consecutively patch tested patients is low and ranges from 0.1% to 0.3% [1, 2, 3]. Only a few cases of ACD to ethylhexylglycerin have been reported, mainly caused by cosmetics, including moisturisers, deodorants and sunscreens [1, 2, 3, 4]. Only one other case of ACD to ethylhexylglycerin not caused by cosmetics has been reported before [8]. In that case, the causative exposures were ultrasonic gels and a lubricating gel. The authors pointed out that these products were considered as medical devices which hampers retrieval of information about their composition [8]. In our case, the culprit disinfectant containing ethylhexylglycerin was registered as a biocidal product and similarly, it was difficult to receive detailed information about its composition. This lack of information and labelling severely impairs allergen avoidance in sensitised individuals.

In conclusion, we here report the second case of ACD to ethylhexylglycerin not caused by cosmetics. It is the first report about occupational ACD to ethylhexylglycerin and the first case related to alcohol‐based hand disinfectants.

## Author Contributions

**Richard Brans:** conceptualization, investigation, writing – original draft, methodology, visualization, writing – review and editing, project administration, data curation. **Christoph Skudlik:** writing – review and editing, resources.

## Consent

Written informed consent was obtained to publish the photograph.

## Conflicts of Interest

The authors declare no conflicts of interest.
